# Pathological Vaginal Discharge among Pregnant Women: Pattern of Occurrence and Association in a Population-Based Survey

**DOI:** 10.1155/2013/590416

**Published:** 2013-06-17

**Authors:** Tânia Maria M. V. da Fonseca, Juraci A. Cesar, Raúl A. Mendoza-Sassi, Elisabeth B. Schmidt

**Affiliations:** ^1^Programa de Pós-Graduação em Educação em Ciências: Química da Vida e Saúde, Universidade Federal do Rio Grande, Rua Dr. Nascimento 737, 96200-300 Rio Grande, RS, Brazil; ^2^Divisão de População & Saúde, Universidade Federal do Rio Grande Área Acadêmica do Hospital Universitário da FURG, Rua Gen. Osório S/N. 96200-190 Rio Grande, RS, Brazil; ^3^Programa de Pós-Graduação em Educação em Ciências: Química da Vida e Saúde, Universidade Federal do Rio Grande, Avenida Itália, km 8, Campus Carreiros, 96203-900 Rio Grande, RS, Brazil

## Abstract

This study aimed to assess the prevalence of pathological vaginal discharge and to describe risk factors associated with pregnant women. All women living in the city of Rio Grande, Southern Brazil, who gave birth in 2010 were included in the study. A standardized questionnaire was administered to collect information on demographic, reproductive, and health-related factors and morbidity during pregnancy. The chi-square test was used to compare proportions, and multivariate Poisson regression with robust variance was performed. Of the 2,395 women studied, 43% had pathological vaginal discharge during pregnancy. The adjusted analysis showed that younger women of lower socioeconomic condition, those with a past history of abortion, vaginal discharge in a previous pregnancy, and treated for depression, anemia, and urinary tract infection during their current pregnancy, were more likely to have pathological vaginal discharge. Vaginal discharge during pregnancy was highly prevalent in the sample studied calling for proper risk factor management at the primary care level.

## 1. Introduction

Vaginal discharge is a common gynecological condition among women of childbearing age that frequently requires care affecting about one-third of all women and half of pregnant women [1–3].

Pathological vaginal discharge can cause serious harm to pregnant women and their children including prematurity, low birth weight, chorioamnionitis, postpartum endometritis, and postcesarean wound infection [4–6]. It is the second leading cause of lost years of healthy life for women aged 15 to 49. It is also a facilitator of human immunodeficiency virus (HIV) infection for the virus can gain entry into cells and for it evidences that these women are having unprotected sex [7].

Vaginal discharge is normal in women in their childbearing years. It derives from physiological secretion of cervical and Bartholin's glands and desquamation of vaginal epithelial cells resulting from bacterial action in the vagina. When abnormal vaginal discharge is more abundant and has an unpleasant odor, it is usually accompanied by vulval or vaginal itching, dysuria, and/or dyspareunia [1, 2]. Studies carried out in developing countries demonstrated that vaginal discharge is caused by sexually transmitted infections (STIs) in up to 90% of cases [8, 9].

During pregnancy genital mucosa becomes thinner and has greater surface area making pregnant women more susceptible to infections [10, 11]. In addition, in Brazil, women in general lack knowledge on this condition, they do not recognize it as being important, and they are not able to follow health providers' guidance to practice good hygiene habits [10, 12]. Brazilian women are also known to use condoms exclusively as a birth control method but not for STI prevention [12, 13].

This study was carried out in Rio Grande, Southern Brazil. This municipality is the only seaport city in the state of Rio Grande do Sul. It has a population of around 200,000 inhabitants (IBGE, 2010) over 2.900 km^2^ and its diverse economy is primarily based on port activities. The city's per capita gross domestic product (GDP) is approximately R$ 32,000. Infant mortality rate of Rio Grande in 2010 was 11.4 per 1,000 live births (2011) [14]. At the time of this study, the city had two hospitals with a total capacity of 600 beds, of which 56 were obstetric beds, and an average of 2,500 deliveries a year [14].

This study aimed to assess the prevalence of pathological vaginal discharge and to describe risk factors associated with pregnant women.

## 2. Methods

A cross-sectional design was used to assess women who gave birth; they were interviewed only once while staying at the maternity wards. This study design was selected because it allows to assess the prevalence of the condition and/or frequent events and to collect information from a large number of individuals in a short time [15].

A precoded questionnaire was developed to collect information on place of residence, demographic characteristics, maternal reproductive history, socioeconomic condition, household assets, housing and sanitation conditions, care during pregnancy and childbirth, and complications during pregnancy. Although most variables are self-explanatory, additional clarification is required for some of them: skin color: categorized by the interviewer as white, mixed, and black; household income: the income of all household members in the month preceding the interview; household asset index: household ownership of goods including vacuum cleaner, washing machine, DVD player, refrigerator, freezer, microwave oven, personal computer, telephone, radio, TV set, motor vehicle, air conditioner; and having a hired household helper. This variable also included maternal education and household income. Different components were generated and the first one was used to create a continuous score that included all variables that make direct contributions. This component had an eigenvalue of 5.18 and explained 32% of the total variance. This variable was subsequently divided into tertiles for the analyses: maternal smoking: if smoked at least once in the six months prior to pregnancy; alcohol use: if used alcohol during pregnancy; occurrence of comorbidities such as anemia, depression and arterial hypertension, among others: affirmative answer if present; perceived abnormal vaginal discharge (outcome): reported vaginal discharge associated with at least one of the following symptoms including itching, dysuria, dyspareunia, odor, and nonwhite color.

Four interviewer candidates were trained for five consecutive days. At the end of training three of them were hired. A pilot study was conducted in the city's two maternity hospitals to test the study questionnaire and check for any issues regarding wording of questions. At this step at least four complete questionnaires were administered by each interviewer. All instruments used in the study were provided with a manual with detailed instructions for completion.

Interviewers coded the complete questionnaires at the end of each data collection day and handed them to the study staff by the end of each week. Open questions were also coded. All questionnaires were fully reviewed and entered in EpiData 3.1. Data was later moved to Stata 11.0 (Stata Corp., College Station, USA) for consistency analysis, categorization of variables, and data analysis.

An analysis was first performed to assess frequencies and consistency of independent variables and the outcome (occurrence of pathological vaginal discharge). Data were entered in a database for labeling and construction of derived variables.

A multivariate analysis was conducted in accordance with a predefined hierarchical model (Table 1). In this model some variables were considered as determinants. The variables included in the first level may have a direct contribution to the outcome (e.g., perceived pathological vaginal discharge during pregnancy) or may have a contribution through variables included in other levels, that is, indirectly contributing to a symptom. Measures of disease occurrence and effect were prevalence and prevalence ratio, respectively. Prevalence ratios (PR) and their crude and adjusted 95% confidence intervals (95% CI) were calculated using Poisson regression with robust adjustment of variance. Variables with *P* < 0.20 were included in the adjusted model to control for potential confounders [16].

The variables included in the first level of the hierarchical model were maternal age, skin color, marital status, maternal education, and socioeconomic condition. In the second level the variables smoking, parity, abortion, and planned pregnancy were included. And in the third level, use of oral contraceptives, prenatal visits, and vaginal discharge in the previous pregnancy were included. To investigate comorbidities associated with the prevalence of vaginal discharge, potential confounding variables that were associated (*P* < 0.20) with the outcome were included. A 95% significance level was used for two-sided tests. In this analysis model, variables at an upper level than the variable in question were considered potential confounders of the outcome while those variables in lower levels were considered potential mediators of the association. Those variables selected at a given level remained in the model and were considered potential risk factors for the outcome even if they lost their statistical significance with the inclusion of lower-level variables.

For quality control 5% of the interviews were repeated. Mothers were randomly selected and home interviews were conducted using a shortened version of the study questionnaire. All respondents signed an informed consent and respondent confidentiality was ensured.

The present study was approved by the Research Ethics Committees of the Universidade Federal do Rio Grande (FURG) (Protocol no. 23116006258/2009-64-53/2009) and of the Santa Casa de Misericórdia de Rio Grande (Protocol no. 009/2009).

## 3. Results 

Of 2,464 pregnant women living in the city of Rio Grande who gave birth in 2010, 2,395 (97.25%) were included in the study.

The prevalence of vaginal discharge during pregnancy was 43% (95% CI 40.9; 44.9) among all women studied. About 20% were adolescents (younger than 20), 68% were white-skinned, 83% lived with a partner, 55% had nine or more years of schooling, 26% were smokers, 44% were primiparous, 14% reported previous abortion, 36% planned pregnancy, 80% attended six or more prenatal visits, and 11% reported pathological vaginal discharge in a previous pregnancy. Only 53% of women who reported the occurrence of vaginal discharge underwent treatment for the symptom.

Table 2 shows the prevalence of pathological vaginal discharge during pregnancy according to the category of the variable included in the model and crude and adjusted PRs. The prevalence of pathological vaginal discharge ranged from 32% in women aged 30 and older to 82% in those who reported pathological vaginal discharge in a previous pregnancy.

The crude analysis showed that pathological vaginal discharge during pregnancy was significantly associated with the following variables: maternal age; living with a partner; household asset index; parity; vaginal discharge in a previous pregnancy; and diabetes, depression, threatened premature labor, urinary infection, and hospitalization during the current pregnancy.

A protective effect of maternal age on the likelihood of occurrence of pathological vaginal discharge was found after adjustment. A PR of 0.72 (95% CI 0.63; 0.82) was found in women aged 30 and more compared to those aged 20 to 29 years. Women in the lower wealth tertile showed a PR of 1.22 (1.07; 1.39) compared to those in the highest tertile. Those who reported a previous abortion and pathological vaginal discharge in a previous pregnancy showed a PR of 1.20 (1.05; 1.38) and 2.26 (2.02; 2.54), respectively, compared to other women. A PR of 1.36 (1.20 to 1.54) and 1.26 (1.15; 1.38) was found in women treated for depression and anemia, respectively, compared to all other women. A PR of 1.27 (1.11; 1.45) was found for threatened premature labor. Finally, a PR of 1.49 (1.36; 1.63) and 1.24 (1.10; 1.41), respectively, was observed on those who had urinary tract infection or were hospitalized.

## 4. Discussion

Vaginal discharge affected 43% of the women studied. The main risk factors associated were young age, low socioeconomic condition, vaginal discharge in a previous pregnancy, depression, anemia, threatened premature labor, urinary infection, and hospitalization in the current pregnancy.

A higher prevalence of vaginal discharge during pregnancy was seen in women younger than 20 (the prevalence ratio was about 50% higher). A number of studies have reported higher prevalence of vaginal discharge symptoms and STDs in younger women [17, 18]. In addition to their greater biological vulnerability, adolescents are more susceptible to engaging in risky behaviors and to acquiring STDs. This is a widely known phenomenon in the context of STDs mostly due to the fact that teenagers believe that love will protect them against STDs, which partly explains the spread of these infections among women with a steady partner [12].

This study found higher rates of vaginal discharge during pregnancy among poor women. Similar results were reported in a study conducted in the same city in 2007 [18].

Reporting vaginal discharge in a previous pregnancy was the strongest predictor of vaginal discharge in the current pregnancy showing a PR twice as high (PR 2.26, 2.02; 2.54). This association was also demonstrated in a 2007 study [17]. It is possible that the previous occurrence and inadequate handling by health providers facilitate the installation of a new infectious process or, by the presence of the same risk factors, this outcome recurs in the next pregnancy. Possibly, it is due to the fact that health providers do not investigate a potential link between the complaint of vaginal discharge and STIs and their mode of transmission, and thus they are not properly managed. The differentiated approach to patients with symptoms suggestive of STIs by health providers increases the probability of treatment of the sexual partner and the return to the service for the control of healing, aspects that are important to break the chain of transmission of STIs, and the recurrence of symptoms [19, 20].

This study found that vaginal discharge was associated with depression; it was seen in 55% of women who reported having depression during pregnancy. Depression rates are higher in women attending gynecological clinics, and qualitative studies have demonstrated a strong relationship between vaginal discharge, psychosomatic symptoms, and stress [21, 22].

Anemia was significantly associated with vaginal discharge during pregnancy. Half of the women who had anemia reported pathological vaginal discharge. This finding is corroborated by another study with pregnant women. The high prevalence of abnormal vaginal discharge in this population shows the need to examine this relationship in greater depth in order to prevent occurrence [23].

Threatened premature labor was significantly associated with pathological vaginal discharge during pregnancy. Just over half (52%) of women who reported threat of preterm labor complained of vaginal discharge. In this study, premature labor occurred in 15% of the women studied. An infectious cause is seen in about 40% of cases of spontaneous premature labor [24, 25]. STIs have been associated with adverse pregnancy outcomes including premature birth and low birth weight—both major determinants of infant morbidity and mortality especially in developing countries where neonatal intensive care is available only in very few centers [6, 25].

This study showed that urinary tract infection reported during pregnancy was strongly associated with vaginal discharge, a finding that is corroborated by other studies [17, 18]. There is growing evidence supporting that genital infections during pregnancy increase the risk of urinary tract infection, which is a major risk factor for premature birth and a leading cause of neonatal death [24, 26]. It clearly shows the importance of early diagnosis and appropriate management of vaginal discharge to reduce infant deaths.

Pathological vaginal discharge was highly prevalent in the population of women studied. Younger women of low socioeconomic condition with a history of several morbid conditions during pregnancy are more likely to have vaginal discharge. These results stress a need for proper management and treatment of vaginal discharge, and pregnant women should be educated on good hygiene habits and prevention. If not, this almost imperceptible though highly prevalent condition among pregnant women will continue to favor the development of more severe conditions that significantly contribute to increased infant mortality. There is an urgent need for intervention.

## Figures and Tables

**Table 1:** Hierarchical model of analysis for factors associated with the occurrence of pathological vaginal discharge.

Level	Variables		
I	*Demographic:*	*Socioeconomic*:	
	Maternal age, skin color, and marital status	Maternal education and household asset index	

II	*Behavioral characteristics*:	*Obstetric history*:	
	Smoking	Parity, previous abortion, and planned pregnancy	

III	*Prior use of contraceptive method*:	*History of morbidities*:	*Current pregnancy care*:
	Oral contraceptives	Vaginal discharge in a previous pregnancy	Prenatal care visits

Outcome	Pathological vaginal discharge during pregnancy		

**Table 2:** Crude and adjusted analysis of factors associated with the prevalence of pathological vaginal discharge during pregnancy. Rio Grande, Brazil, 2010 (*N* = 2,395).

Level	Variable	Vaginal discharge (%)	Crude analysis		Adjusted analysis	
			Prevalence ratio (95% CI)	*P* value	Prevalence ratio (95% CI)	*P* value
I	Age (years)			<0.001		<0.001*
	Up to 19	49.9	1.08 (0.96; 1.20)		1.05 (0.94; 1.18)	
	20–29	46.3	1.00		1.00	
	30 or more	32.1	0.70 (0.61; 0.78)		0.72 (0.63; 0.82)	
	Skin color			0.44		0.44
	White	42.5	1.00		1.00	
	Mixed	45.2	1.06 (0.95; 1.19)		1.02 (0.91; 1.14)	
	Black	40.8	0.96 (0.82; 1.13)		0.91 (0.77; 1.07)	
	Living with a partner			0.01		0.11
	No	48.8	1.18 (1.05; 1.31)		1.10 (0.98; 1.23)	
	Yes	41.7	1.00		1.00	
	Education (years of schooling)			0.17		0.78
	0–4	40.3	1.09 (0.86; 1.38)		0.93 (0.72; 1.19)	
	5–8	44.8	1.21 (1.01; 1.44)		0.97 (0.80; 1.18)	
	9–11	43.1	1.16 (0.97; 1.38)		1.01 (0.85; 1.22)	
	12 or more	37.1	1.00		1.00	
	Household asset index (tertiles)			<0.001		0.004
	Poorest	48.8	1.28 (1.14; 1.44)		1.22 (1.07; 1.39)	
	Intermediate	41.9	1.10 (0.98; 1.24)		1.05 (0.92; 1.19)	
	Richest	38.0	1.00		1.00	

II	Smoking in the six months prior to pregnancy			0.48		0.80
	No	42.5	1.00		1.00	
	Yes	44.1	1.04 (0.94; 1.15)		0.99 (0.89; 1.10)	
	Parity			0.01		0.02
	0	45.4	1.22 (1.07; 1.40)		1.20 (1.02; 1.40)	
	1	38.4	1.03 (0.86; 1.23)		1.02 (0.85; 1.22)	
	2	45.6	1.22 (1.05; 1.42)		1.22 (1.05; 1.42)	
	3 and more	37.2	1.00		1.00	
	Abortion			0.22		0.01
	No	42.4	1.00		1.00	
	Yes	45.9	1.08 (0.95; 1.23)		1.20 (1.05; 1.38)	
	Planned pregnancy			0.08		0.28
	Yes	40.1	1.00		1.00	
	More or less	41.7	1.04 (0.87; 1.25)		1.01 (0.85; 1.21)	
	No	44.8	1.12 (1.01; 1.24)		1.09 (0.98; 1.21)	
	Oral contraceptives			0.74		0.41
	No	43.7	1.00		1.00	
	Yes	42.7	0.98 (0.86; 1.11)		1.06 (0.93; 1.21)	

III	At least 6 prenatal visits			0.46		0.61
	No	44.9	1.04 (0.93; 1.17)		0.97 (0.86; 1.09)	
	Yes	43.0	1.00		1.00	
	Vaginal discharge in a previous pregnancy			<0.001		<0.001
	No	40.1	1.00		1.00	
	Yes	84.5	2.45 (2.20; 2.73)		2.26 (2.02; 2.54)	
	High blood pressure			0.61		0.37
	No	42.6	1.00		1.00	
	Yes	43.9	1.03 (0.92; 1.16)		1.05 (0.94; 1.18)	
	Diabetes			0.66		0.39
	No	42.8	1.00		1.00	
	Yes	45.3	1.06 (0.82; 1.36)		1.11 (0.87; 1.42)	
	Depression			<0.001		<0.001
	No	41.4	1.00		1.00	
	Yes	55.5	1.34 (1.19; 1.52)		1.36 (1.20; 1.54)	
	Anemia			<0.001		<0.001
	No	38.3	1.00		1.00	
	Yes	49.3	1.29 (1.17; 1.41)		1.26 (1.15; 1.38)	
	Threatened abortion			0.13		0.10
	No	42.5	1.00		1.00	
	Yes	48.6	1.14 (0.96; 1.36)		1.16 (0.97; 1.38)	
	Threatened premature labor			0.002		<0.001
	No	42.0	1.00		1.00	
	Yes	52.1	1.24 (1.08; 1.42)		1.27 (1.11; 1.45)	
	Urinary infection			<0.001		<0.001
	No	35.4	1.00		1.00	
	Yes	54.2	1.53 (1.40; 1.68)		1.49 (1.36; 1.63)	
	Hospitalization			<0.001		0.001
	No	41.7	1.00		1.00	
	Yes	52.9	1.27 (1.12; 1.44)		1.24 (1.10; 1.41)	

*Adjustment for maternal age, marital status, socioeconomic condition, parity and previous abortion.
